# Factors Influencing the Size of a Non-Traumatic Full-Thickness Rotator Cuff Tear: Focusing on Socioeconomic Factors

**DOI:** 10.3390/ijerph19106137

**Published:** 2022-05-18

**Authors:** Suk-Woong Kang, Chan-Kue Park, Seung-Hun Woo, Tae-Woo Kim, Min-Hui Moon, Ji-Hee Yang, Min-Hyeok Choi

**Affiliations:** 1 Department of Orthopedic Surgery, Pusan National University Yangsan Hospital, Yangsan 50612, Korea; osksw98@pusan.ac.kr (S.-W.K.); shwoo@pusan.ac.kr (S.-H.W.); tw1904@pusan.ac.kr (T.-W.K.); 2 Department of Orthopedic Surgery, Medical College, Pusan National University, Yangsan 50612, Korea; 3 Department of Radiology, Pusan National University Yangsan Hospital, Medical College, Pusan National University, Yangsan 50612, Korea; julias815@pusan.ac.kr; 4 Office of Public Healthcare Service, Pusan National University Yangsan Hospital, Yangsan 50612, Korea; mhmoon@pnuyh.co.kr; 5 Department of Medicine, Medical College, Pusan National University, Yangsan 50612, Korea; zzzwlgml159@pusan.ac.kr; 6 Department of Preventive and Occupational & Environmental Medicine, Medical College, Pusan National University, Yangsan 50612, Korea

**Keywords:** musculoskeletal disorders, rotator cuff tear, tear size, risk factors, socioeconomic factors

## Abstract

This study aimed to identify the risk factors for non-traumatic rotator cuff tears in Korean adult patients who underwent surgical treatment, focusing on socioeconomic factors. A retrospective study was conducted with 659 patients who were diagnosed with a full rotator cuff tear and underwent surgical treatment. The outcome variable was the rotator cuff tear size (mm), as indicated by preoperative magnetic resonance imaging. Socioeconomic variables included occupation, education level, insurance type, and residential area. Univariate analyses were used to evaluate the relation between tear size and independent variables, and multivariate regression was used to estimate the effects of socioeconomic factors on tear size after adjusting for other variables. Significant differences were found in mean tear size according to age, occupation, residence area, and symptom duration (*p* < 0.05) in multivariate regression analysis. Rural residents had a 2.12 mm larger tear size than urban residents. Compared to National Health Insurance patients, the tear size of Medicaid beneficiaries was significantly larger (6.79 mm) in urban areas. The larger the rotator cuff tear, the greater the risk of retear and poor shoulder function. Therefore, policy efforts are required to expand access to medical care for the vulnerable.

## 1. Introduction

Rotator cuff tears are among the most common upper extremity diseases, and the frequency of surgical treatment has significantly increased in recent years [1,2,3]. Various studies on the risk factors for rotator cuff tears have identified age, diabetes, hyperlipidemia, body mass index (BMI), smoking, and occupational history as factors associated with rotator cuff tears [4,5,6].

The size of the rotator cuff tear is important. Many studies have been conducted on the relationship between retear rates and the size of rotator cuff tear after surgery. In addition, the risk of pain development is high in patients with full tears and tear enlargements in those with asymptomatic rotator cuff tears, even with conservative treatment [7]. Moreover, a significant decrease in shoulder strength is observed as the size of the rotator cuff tear increased [8]. Therefore, it is important to study the factors affecting rotator cuff size. In general, surgical treatment shows good results in symptomatic rotator cuff tears. However, in some patients, revision surgery is performed because of retear, or there are cases where the function is not good. In many previous studies, age and tear size are considered the most influential factors in retears after rotator cuff repair. In a systematic review study, the average retear rate of patients with small- to medium-sized rotator cuff tears was 12.5%, compared to an average of 37% in large to massive rotator cuff tear patients [9,10,11]. A recent systematic review reported that age, BMI, fatty infiltration, symptom duration, and tear size are risk factors for retear [11]. Multivariate analysis revealed that age, initial tear size, and fatty degeneration were the independent risk factors [12]. Moreover, rotator cuff tear size increase with time [13]. Tsuchiya et al. reported an increase in rotator cuff tears regardless of symptoms [14]. According to Yamamoto et al., the size of a symptomatic rotator cuff tear progresses to 47% of the shoulder for an average of 19 months, and the rate of progression is 3.8 mm/year in length and 2.0 mm/year in width [15]. They further identified medium-sized full tears and smoking as the risk factors for tear progression.

Many studies have linked musculoskeletal diseases to socioeconomic factors [16,17,18]. Shoulder lesions tend to be reported by workers in physically demanding jobs. Shoulder lesions are also associated with heavy lifting, awkward posture, and vibration [19,20,21]. Monrad et al. found a 1.4-fold higher mean incidence rate in rural areas, where there was a greater occurrence of manual labor than in urban areas [22]. Hagen et al. reported that the risk of chronic musculoskeletal complaints was 1.8-fold higher in the group with a low-income level than in the group with a higher-income level [23]. Moreover, Hong and Kim’s study in Korea revealed that Medicaid beneficiaries had a 1.56 times higher arthritis prevalence than National Health Insurance (NHI) beneficiaries [24].

Although some studies have been conducted on the association between rotator cuff size and work relevance, there are few studies on other socioeconomic factors [2,11,25], particularly regarding residence area and medical insurance type. Thus, our study aimed to identify the factors that influence the size of non-traumatic full-thickness rotator cuff tears in patients who had undergone surgical treatment, and determine whether socioeconomic factors are related to tear size.

## 2. Materials and Methods

### 2.1. Study Population

We conducted a retrospective study of 876 Korean adult patients diagnosed with non-traumatic rotator cuff tears (International Statistical Classification of Diseases and Related Health Problems 10th Revision code: M75.1) and underwent surgical treatment between March 2010 and March 2020. In patients with symptomatic rotator cuff rupture, surgical treatment was performed for those who did not improve after conservative treatment such as drug treatment, subacromial steroid injection, and physical therapy for at least 3–6 months. A traumatic tear was considered a traumatic event when there was a definite trauma history to the shoulder joint and magnetic resonance imaging (MRI) revealed a signal change suggesting trauma, such as a bone bruise, hemarthrosis, and edema in the rotator cuff tendon or muscle [26]. Debridement, synovectomy, and bursectomy were performed for less than 50% of rotator partial tears during surgery, and acromioplasty was performed for bursal-side tears with acromial spur and coracoacromial ligament fraying. For more than 50% of partial rotator tears, rotator cuff repair was performed after complete full-tear conversion [27].

**Figure 1:**
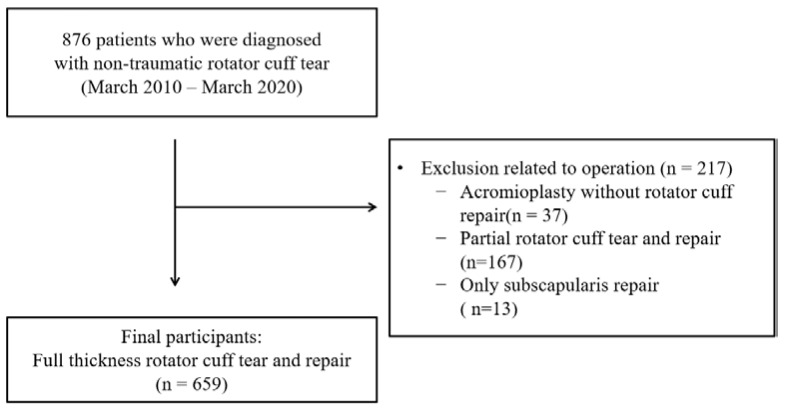
Diagram of the participants.

Inclusion criteria of this study were patients with non-traumatic full thickness rotator cuff tears who underwent rotator cuff repair. It was decided to exclude cases where it was difficult to accurately measure the size of a rotator cuff tear, so the exclusion criteria are as follows: (1) patients who had undergone acromioplasty for partial rotator cuff tears (37 patients); (2) patients undergone rotator cuff repair for rotator cuff tears (167 patients); (3) patients undergone only subscapular repair (13 patients). Therefore, data from 659 patients who underwent arthroscopic repair for non-traumatic full thickness rotator cuff tears were used (Figure 1).

### 2.2. Variables

Preoperative MRI was performed in all patients, and surgery was determined. The outcome variable was the tear size of the rotator cuff, as confirmed by preoperative MRI. The tear size, reported in 1 mm units, was measured by a shoulder orthopedic radiologist with more than 10 years of experience. Shoulder MRI was performed using 1.5-T (MAGNETOM Avanto, Siemens Healthinneers) and 3-T (MAGNETOM Verio, Skyra, and VIDA, Siemens Healthinneers) MRI systems that included axial, oblique coronal, and oblique sagittal T1-weighted and fat-suppressed T2-weighted fast spin-echo sequences (FSE). The supraspinatus, infraspinatus, and subscapularis tendons were evaluated separately. Cuff tear was defined as any discontinuity of the tendon fibers or an intratendinous fluid-equivalent signal intensity lesion. To measure the tear size of the supraspinatus or infraspinatus tendon complex, the mediolateral dimension on coronal oblique fat-suppressed T2-weighted FSE and anteroposterior dimension on sagittal oblique fat-suppressed T2-weighted FSE were referred to. The tear size was determined as the greater of the two values [28].

Variables affecting non-traumatic rotator cuff tears size were selected by reviewing the literature [17,18,20]. For the biological- and comorbidity-related independent variables, sex, age (30–49, 50–69, and ≥70 years), obesity (BMI ≥ 25 kg/m^2^), diabetes (fasting blood sugar level ≥ 126 mg/dL or diabetes medication), and duration (years) of symptoms, such as shoulder pain or mobility restriction, were adopted. Socioeconomic variables included occupation (non-manual and manual labor), education level (≤middle school and ≥high school), insurance type (NHI and Medicaid), and area of residence (urban and rural).

Occupations were classified based on the current job if currently employed, and the longest job held if retired. Housewives were included in the non-manual labor group. Most Koreans are enrolled in the NHI, and the insurance fund is operated by the Korean government. People belonging to the NHI pay only a part of the hospitalization and surgery costs. Meanwhile, people with a low socioeconomic status are enrolled in the Medicaid system. Medicaid beneficiaries accounted for 2.9% of the total Korean population in 2019 [29]. As such, insurance type is an indicator of socioeconomic level in Korea, along with basic livelihood welfare benefits, and is often used as a factor variable in studies on health inequality [18,30,31]. Regarding the area of residence, *dong* addresses were classified as urban and *eup* and *myeon* addresses as rural. This categorization has previously been used in research on the health disparity between urban and rural areas and the impact of these areas on health [32,33].

### 2.3. Statistical Analyses

The mean and standard deviation of tear size were calculated according to the category of each factor. Univariate analysis was used to evaluate the relation between non-traumatic rotator cuff tear size and independent variables. A two-sided Student’s *t*-test was performed to evaluate differences in the mean rotator cuff tear size according to sex, education level, area of residence, insurance type, obesity, occupation, and diabetes. Analysis of variance was used to determine the influence of age group. Univariate regression analysis was conducted to evaluate the effect of symptom duration on tear size.

The effects of the independent factors on tear size were then estimated after adjusting for the influence of other confounding variables using multivariate regression analysis. Variables to be included in the multivariate regression model were selected based on the results of previous studies and univariate analyses. The effect size of each variable was determined through the coefficient and *p*-value. Multivariate regression analyses stratified into rural and urban areas were performed to determine the association between these factors and rotator cuff tear size. Statistical significance was set at *p* < 0.05. All statistical analyses were performed using STATA MP 17.0 (Stata Corporation, College Station, TX, USA).

### 2.4. Ethical Considerations

This study was approved by the Institutional Review Board of Pusan National University Yangsan Hospital (IRB No. 05-2021-185).

## 3. Results

Table 1 presents the general characteristics of the participants. The mean age of the patients was 63.0 ± 8.0 years, mean symptom duration was 1.7 ± 1.8 years, and mean tear size was 25.4 ± 13.1 mm.

Table 2 shows the mean and standard deviation of tear size by rotator cuff injury, according to these factors. A significant difference was observed in the mean tear size according to age, occupation, educational level, area of residence, insurance type, and symptom duration (*p* < 0.05). The mean tear size by age group was largest at 70 years and over (28.0 mm) and smallest in those aged 30–49 years (14.8 mm), and the difference was statistically significant (*p* < 0.001). Patients engaged in manual labor had a larger mean tear size than those engaged in non-manual labor (*p* < 0.001). The mean tear size was also greater in groups with lower educational levels (≤middle school), residents of rural areas, or among Medicaid beneficiaries. Moreover, as the symptom duration increased by one year, the tear size significantly increased by 0.96 mm (*p* = 0.001).

Table 3 indicates the results of the multivariate regression analysis of the influence of these factors on tear size due to rotator cuff injury in all patients. Age, occupation, residence area, and symptom duration significantly affected tear size. Patients in the older age groups had significantly larger tear sizes than those aged 30–49 years. The group engaged in manual labor had a tear size larger by 2.73 mm (95% confidence interval [CI]: 0.89–4.90, *p* = 0.013) than non-manual laborers after controlling for the impact of other variables. Similarly, rural residents showed larger tear sizes (2.12 mm, 95% CI: 0.03–4.22, *p* = 0.047) than urban residents, with the influence of other factors controlled. Moreover, as the symptom duration increased by one year, the tear size significantly increased (0.74 mm, 95% CI: 0.17–1.32, *p* = 0.012).

Table 4 presents the influence of the factors on non-traumatic rotator cuff tear size by residence area. In rural areas, the factors that showed a significant impact on the tear size of the rotator cuff were age group and symptom duration. The tear size in the oldest age group was significantly larger than that in the youngest group. Tear size significantly increased as the symptom duration increased by one year (1.75 mm, 95% CI: 0.14–3.37, *p* = 0.034), with the influence of other factors adjusted. In urban areas, factors with a significant impact on rotator cuff tear size were age group, occupation, and insurance type. The two older age groups reported larger tear sizes than the younger age group. Moreover, the manual labor group had a larger tear size (2.95 mm, 95% CI: 0.40–5.49, *p* = 0.023) than the non-manual group. Compared with the group covered by NHI, Medicaid beneficiaries had significantly larger tear sizes (6.79 mm, 95% CI: 1.33–12.25, *p* = 0.015).

## 4. Discussion

In this study, the patients who underwent surgical treatment for full-thickness non-traumatic rotator cuff tears had a mean age of 63.0 ± 8.0 years, mean tear size of 25.4 ± 13.1 mm, and mean symptom duration of 1.7 ± 1.8 years. Univariate analysis identified risk factors related to tear size, such as age, occupation, education level, residential area, insurance type, and symptom duration. In the multivariate regression analysis, older age, manual labor, and rural residence were associated with a larger tear size after controlling for the influence of other factors. Symptom duration was also a risk factor for larger tear size.

Rotator cuff tear is one of the most common diseases in orthopedics and accounts for the largest proportion of shoulder diseases. In addition, rotator cuff tear is functionally degraded and is considered a progressive disease [8]. Therefore, several studies have been conducted on this topic. In particular, there are many studies related to re-rupture and functional deterioration after the surgical treatment of rotator cuff tears. However, few studies have focused on the factors affecting the size of the rotator cuff tear before rotator cuff surgery, and in particular, no studies have paid attention to the socioeconomic level to the best of the authors’ knowledge. The size of the rotator cuff tear causes functional problems as well as surgical outcomes and affects the course of the disease. This study could be the first to investigate the factors related to the size of the preoperative rotator cuff tear, including the socioeconomic level, in patients who underwent surgical treatment in a hospital.

Many studies have mentioned that the size of the rotator cuff tear in elderly patients is a risk factor for retear. A retear rate of 10–48% has been reported in a systemic review study [7]. In another study, the average retear rate in patients with small-to-medium rotator cuff tears was 12.5%, compared to 37% in patients with large rotator cuff tears [9,10]. Therefore, larger rotator cuff tears are a major concern for orthopedic surgeons. As the size of a rotator cuff tear has a significant effect on the outcome of surgical treatment, early detection is considered important, and surgical treatment should be performed at an appropriate time. Moreover, rotator cuff tears increase in size over time. Therefore, scholars have investigated the degree of rotator cuff tear progression. Yamamoto et al. reported that the tear size of symptomatic rotator cuff tears progresses in 47% of the shoulder at a rate of 3.8 mm/year in length and 2.0 mm/year in width [15]. Maman et al. found that, in full-thickness rotator cuff tear cases, 52% of patients showed an increase in tear size of 2 mm or more [34]. They also found that the longer the follow-up period and greater the age, the greater the tear size. In one prospective study, the size of a full-thickness rotator tear tended to increase significantly, with 18% of full-thickness tears showing an increase of >5 mm and 40% of partial-thickness tears progressing to the full-thickness type. The limitations of these studies include a small number of patients and relatively short follow-up period (e.g., X months). In this study, it was found that as the duration of symptoms increased by one year, the tears increased by 0.74 mm, after controlling for the influence of other factors. Although the size increase in individual patients could not be directly studied, the results indirectly reflected the effect of X months on the follow-up period in a relatively large number of patients.

This work fills a gap in the research on socioeconomic risk factors for rotator cuff tears, which remains limited. Park et al. reported a high prevalence of rotator cuff tears in manual workers [35]. In 2004–2006, the results of a multivariate analysis in a cohort study of 393 asymptomatic full-thickness rotator cuff tear patients indicated that pain increased with increasing comorbidities, lower education levels, and non-white race [30]. Another study found that older, higher comorbidity, female, non-white, and dual-eligible Medicaid patients underwent significantly fewer surgeries. If surgical treatment is delayed in these patients, the outcome of the surgical treatment may be poor owing to the increase in tear size. In this study, univariate analysis showed an increase in tear size according to manual labor employment, educational level, residence area, and insurance type. The multivariate analysis clarified that tear size was greater in older patients, in manual laborers, and in patients in rural areas. In a comparison between urban and rural residents, tear size increased in both areas as age increased. In particular, tear size was related to symptom duration in rural residents, Medicaid beneficiaries, and manual labor employment in urban residents.

Residents in rural areas reportedly experience more musculoskeletal disorders owing to an aging population and limited access to healthcare [18,32]. Indeed, our study showed that the size of non-traumatic rotator cuff tears was also larger in rural residents, and was found to be statistically significant even after adjusting for other factors such as age. Gupta identified a high prevalence of lower back, knee, and shoulder pain among musculoskeletal disorders in farmers, that may be attributed to poor posture and lack of ergonomic awareness [36]. Based on the results of previous research and our study, rural residents would benefit from the early detection of rotator cuff tears, whereas for urban residents, priority should be given to the formulation of policies for improving access to medical care among persons with low socioeconomic status or manual labor employment. In Korea, a national health screening program is being implemented. The screening includes height, weight, blood pressure, glucose level, basic blood and urine tests, and chest radiography, but does not include musculoskeletal ultrasonography [37]. Han’s study on the rate of national screening revealed that urban residents were 1.42 times more likely to receive health screenings than rural residents [38]. Based on previous studies and the results of this study, it is necessary to include items (e.g., ultrasonography) for early detection of musculoskeletal disorders such as rotator cuff tears in the national health examination program. In addition, if resources are limited, a policy of providing vouchers for examinations for the elderly, rural area residents, and manual laborers should be considered.

This study had several limitations. First, a cross-sectional study of patients who underwent surgical treatment was conducted. Therefore, the prevalence and progression of the cuff tears could not be determined. Second, tear size could not completely replace severity as an indicator of rotator cuff tears. Future studies should consider other factors such as arthritis, biceps injury, muscle atrophy, and labral tears. Third, patients who did not undergo surgical treatment were not included for comparison with the current sample results. A comparative analysis could shed light on the influence of surgical treatment on study factors. Fourth, although symptom duration is related to tear size, it is impossible to know when the tear actually started because symptom duration is subjective. However, because patients who underwent surgery at this hospital for 10 years were included, the results are reliable. Finally, the level of physical activity is an important factor influencing musculoskeletal diseases. The variables for physical activity level in daily life could not be used because of the limitations of the data source.

## 5. Conclusions

The age and duration of symptoms significantly affected the size of rotator cuff tears, as did socioeconomic factors such as insurance type, residence area, and type of occupation. The larger the rotator cuff tear, the greater the risk of retear and poor shoulder function. Therefore, socioeconomically vulnerable patients may have a higher severity of non-traumatic rotator cuff tears and a greater risk of postoperative complications. Policy efforts are required to expand access to medical care for vulnerable people.

## Figures and Tables

**Table 1:** General characteristics of the participants.

		N	%
**Total**		659	100.0
Sex	Female	290	44.0
	Male	369	56.0
Age ^1^ (years)		63.0 ± 8.0 (36–87, 63, 12)	
Age group (years)	30–49	21	3.2
	50–69	491	74.5
	≥70	147	22.3
Occupation	Non-manual	410	62.2
	Manual	249	37.8
Education	≤Middle school	352	53.4
	≥High school	307	46.6
Area of residence	Urban	437	66.3
	Rural	222	33.7
Insurance type	National Health Insurance (NHI)	630	95.6
	Medicaid	29	4.4
Obesity (BMI ≥ 25 kg/m^2^)	No	348	52.8
	Yes	311	47.2
Diabetes	No	555	84.2
	Yes	104	15.8
Symptom duration ^1^ (year)		1.7 ± 1.8 (1–10, 1, 1)	
Tear size ^1^ (mm)		25.4 ± 13.1 (5–64, 22, 21)	

^1^ Mean ± standard deviation (minimum value–maximum value, median, interquartile range).

**Table 2:** Comparison of the non-traumatic full thickness rotator cuff tear sizes (mm) using univariate analysis ^1^.

		Mean	Median	SD ^2^	IQR ^2^	*p*-Value
Sex	Female	25.5	22.8	13.2	21.0	0.864
	Male	25.3	21.0	13.1	21.0	
Age group (years)	30–49	14.8	13.0	7.5	8.0	<0.001
	50–69	25.1	21.0	13.3	20.0	
	≥70	28.0	29.0	12.5	24.0	
Occupation	Non-manual	24.0	20.0	12.8	20.0	<0.001
	Manual	27.7	26.0	13.4	23.0	
Education	≤Middle school	27.1	25.0	13.1	23.0	<0.001
	≥High school	23.5	20.0	12.9	19.0	
Area of residence	Urban	24.7	21.0	12.6	20.0	0.042
	Rural	26.9	24.0	14.1	25.0	
Insurance type	NHI	25.1	21.0	13.1	21.0	0.007
	Medicaid	31.8	36.0	13.0	20.0	
Obesity	No	25.2	22.0	13.0	22.0	0.649
	Yes	25.6	22.0	13.3	21.0	
Diabetes	No	25.4	21.0	13.4	22.0	0.910
	Yes	25.5	25.0	12.0	19.5	
Symptom duration ^3^		1.96 (0.39–1.53)				0.001

^1^ Student’s *t*-test for sex, education, area of residence, insurance type, obesity, occupation, and diabetes; analysis of variance for age group; univariate regression analysis for symptom duration. ^2^ SD: standard deviation; IQR: interquartile range. ^3^ Coefficient (β), 95% confidence interval of coefficient and *p*-value using univariate regression analysis.

**Table 3:** Results of multivariate regression analysis on the influence of factors on the size of the non-traumatic full thickness rotator cuff tears.

		Coefficient	(95% CI) ^1^	*p*-Value
Sex	Female	Reference		
	Male	−0.25	(−2.39–1.88)	0.815
Age group (years)	30–49	Reference		
	50–69	8.38	(2.65–14.11)	0.004
	≥70	9.95	(3.78–16.13)	0.002
Occupation	Non-manual	Reference		
	Manual	2.73	(0.89–4.90)	0.013
Education	≥High school	Reference		
	≤Middle school	2.00	(−0.16–4.16)	0.070
Area of residence	Urban	Reference		
	Rural	2.12	(0.03–4.22)	0.047
Insurance type	NHI	Reference		
	Medicaid	4.42	(−0.55–9.4)	0.081
Obesity	No	Reference		
	Yes	1.04	(−0.94–3.01)	0.302
Diabetes	No	Reference		
	Yes	−1.29	(−4.03–1.44)	0.354
Symptom duration		0.74	(0.16–1.31)	0.012

^1^ Coefficient (95% CI): coefficient of variable and 95% confidence interval.

**Table 4:** Influence of factors on the size of the rotator cuff tear according to urban and rural residence.

		Rural			Urban		
		Coefficient	(95% CI) ^1^	*p*-Value	Coefficient	(95% CI) ^1^	*p*-Value
Sex	Female	Reference			Reference		
	Male	−0.97	(−5.09–3.15)	0.644	−0.01	(−2.52–2.49)	0.991
Age group (years)	30–49	Reference			Reference		
	50–69	9.87	(−1.03–20.77)	0.076	7.65	(0.92–14.39)	0.026
	≥70	11.59	(0.02–23.19)	0.047	9.09	(1.77–16.4)	0.015
Occupation	Non-manual	Reference			Reference		
	Manual	2.25	(−1.88–6.39)	0.284	2.95	(0.40–5.49)	0.023
Education	≥High school	Reference			Reference		
	≤Middle school	2.44	(−1.68–6.55)	0.245	1.71	(−0.84–4.26)	0.188
Insurance type	NHI	Reference			Reference		
	Medicaid	−2.95	(−14.46–8.57)	0.615	6.79	(1.33–12.25)	0.015
Obesity	No	Reference			Reference		
	Yes	0.93	(−2.81–4.67)	0.624	1.02	(−1.3–3.35)	0.387
Diabetes	No	Reference			Reference		
	Yes	−1.53	(−6.98–3.92)	0.582	−1.01	(−4.21–2.19)	0.535
Symptom duration		1.75	(0.14–3.37)	0.034	0.52	(−0.09–1.12)	0.094

^1^ Coefficient (95% CI): coefficient of variable and 95% confidence interval.
